# SENP3 grants tight junction integrity and cytoskeleton architecture in mouse Sertoli cells

**DOI:** 10.18632/oncotarget.16915

**Published:** 2017-04-07

**Authors:** Di Wu, Chun-Jie Huang, Faheem Ahmed Khan, Xiao-Fei Jiao, Xiao-Ming Liu, Nuruliarizki Shinta Pandupuspitasari, Rahim Dad Brohi, Li-Jun Huo

**Affiliations:** ^1^ Key Laboratory of Agricultural Animal Genetics, Breeding and Reproduction of Ministry of Education, College of Animal Science and Technology, Huazhong Agricultural University, Wuhan 430070, Hubei, China; ^2^ Second Affiliated Hospital and Center of Reproductive Medicine, The Second Affiliated Hospital & Yuying Children's Hospital, Wenzhou Medical University, Wenzhou 325000, Zhejiang, China; ^3^ The Center for Biomedical Research, Tongji Hospital, Tongji Medical College, Huazhong University of Science and Technology, Wuhan 430070, Hubei, China

**Keywords:** SUMOylation, Sertoli cell, tight junction, cytoskeleton, blood-testis barrier

## Abstract

Germ cells develop in a sophisticated immune privileged microenvironment provided by specialized junctions contiguous the basement membrane of the adjacent Sertoli cells that constituted the blood-testis barrier (BTB) in seminiferous epithelium of testis in mammals. Deciphering the molecular regulatory machinery of BTB activity is central to improve male fertility and the role of post-translational modification including SUMOylation pathway is one of the key factors. Herein, we unveiled the mystery of the SUMO-2/3 specific protease SENP3 (Sentrin-specific protease 3) in BTB dynamics regulation. SENP3 is predominantly expressed in the nucleus of Sertoli and spermatocyte cells in adult mouse testis, and knockdown of SENP3 compromises tight junction in Sertoli cells by destructing the permeability function with a concomitant decline in trans-epithelial electrical resistance in primary Sertoli cells, which could attribute to the conspicuous dysfunction of tight junction (TJ) proteins (e.g., ZO-1, occludin) at the cell-cell interface due to the inactivation of STAT3. Moreover, SENP3 knockdown disrupts F-actin architecture in Sertoli cells through intervening Rac1/CDC42-N-WASP-Arp2/3 signaling pathway and Profilin-1 abundance. Our study pinpoints SENP3 might be a novel determinant of multiple pathways governing BTB dynamics in testis to support germ cells development in mammals.

## INTRODUCTION

Spermatogenesis is an inherently complicated intricate process that produce the male germ cells termed sperms and thereby holds the heart of male fertility. Spermatogenesis depends on the promiscuous cross-talk between somatic cells and developing germ cells under the concerted action of a coordinated regulatory network composing of endocrine, paracrine, and autocrine factors [1, 2]. Seminiferous tubule, the testicles functional unit is consist of Sertoli and spermatogenic cells, yields 10-40 million spermatozoa per day among mammalian species once they reach the stage of puberty [1]. Therefore, understanding the molecular mechanisms by which harnessing spermatogenesis will be of great medical and societal significance to provide novel implications for the treatment of male infertility and/or its related unwanted defects.

Predisposing of spermatogenic cells into the risk of infectious and/or host immune response is finely erased by the microenvironment created by blood-testis barrier (BTB). BTB is anatomically constituted of tight junction (TJ) composing of co-existing basal ectoplasmic specializations (basal ES), gap junction (GJ) and desmosome between adjacent Sertoli cells, which are near the basement membrane of seminiferous epithelium that partitions seminiferous epithelium into the basal and apical compartments [3]. The movement of preleptotene spermatocytes in immune privileged microenvironment without compromising its integrity in adult testes is supported by extensive restructuring of specialized junction between Sertoli-Sertoli cells [4]. BTB dynamics have been reported to be regulated by a spectrum of cytokines and testosterone which orchestrate actin architecture and the function of integral membrane proteins via MAPK signaling cascade [5, 6, 7, 8]. Moreover, the actin cytoskeletal activity endowed by actin binding proteins such as Arp2/3 complex, Wiskott-Aldrich syndrome protein (WASP) family, and actin regulator proteins such as non-receptor protein tyrosine kinases (e.g., FAK) and GTPase (e.g., Rac1 and CDC42) in Sertoli cells is also pivotal for BTB dynamics [9, 10, 11, 12]. The mechanisms underlying the regulation of BTB permeability and its participating molecules nevertheless remain largely elusive.

SUMOylation has been in the limelight as a critical regulator of numerous cellular processes [13, 14, 15, 16]. Notably, all the three SUMO isoforms (SUMO-1, SUMO-2 and SUMO-3) have been implicated in human spermatogenesis, which is supported by the findings that human patients with disrupted SUMOylation status experience the symptoms of impaired spermatogenesis [17]. The reversible dynamics of SUMOylation is conferred by the canonically termed Sentrin/SUMO-specific proteases (SENPs, SENP1-3 and SENP5-7 in mammals) [18, 19]. SENP3 has propensity activities to mature SUMO-2/3 and de-conjugate it from a spectrum of targets including multiple transcription factors such as STAT3 and thereby regulates the expression profile of respective target gene [20, 21]. Significantly indeed, SENP3 could boost the transcriptional activity of STAT3 by relaxing SUMO-2/3-ylation on STAT3 [21, 22]. In testis, spermatogonia renewal, meiotic cell progression, spermiogenesis and spermiation are intimately related to the underlying actin-based cytoskeleton architecture [4, 23]. More importantly, actin-binding proteins and actin regulatory proteins are emerging as new categories of SUMO substrates [24, 25, 26]. Previously, we determined the role of SENP3 in conferring microtubule architecture during mouse oocyte meiosis [27]. However, it is still remains unknown whether SENP3 contributes to BTB dynamics and, if so, what is the underlying mechanism that it might involve in?

In this study, we found SENP3 predominantly expressed in the nucleus of Sertoli cells and spermatocyte in adult mouse testis. More importantly, knockdown of SENP3 perturbed the TJ permeability function and compromised the trans-epithelial membrane resistance by attenuating distribution of TJ proteins at the cell-cell interface and F-actin organization. Our study extends the knowledge dimension of SENP3 by determining its novel relevance to mammalian spermatogenesis partially by intervening TJ and F-actin organization.

## RESULTS

### SENP3 is expressed by Sertoli and germ cells in mouse testis

**Figure 1:**
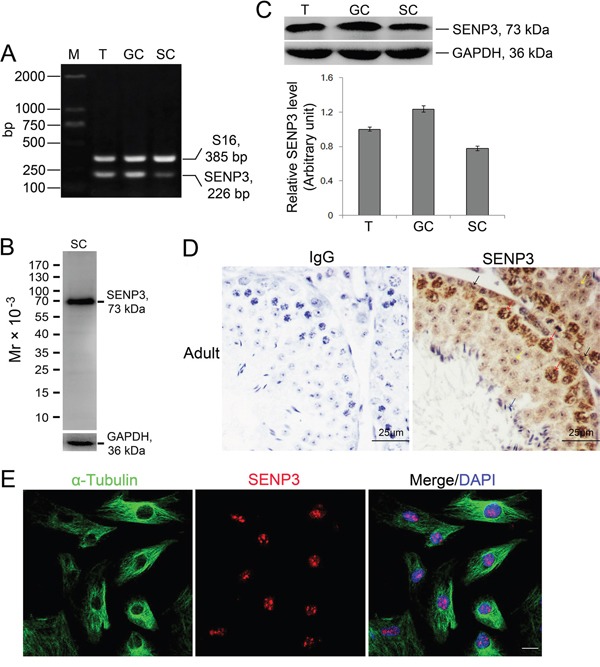
Expression dynamics of SENP3 in Sertoli and germ cells in mouse testis **(A)** Expression of SENP3 in adult mouse testis (T), germ cells (GC) and Sertoli cells (SC) determined by RT-PCR assay using primer specific to *senp3*, and S16 served as a loading PCR control (Supplementary Table 2). M, DNA markers in base pair (bp). **(B)** Specificity of anti-SENP3 antibody was determined by immunoblot analysis using lysate of Sertoli cells with GAPDH severed as the loading control. **(C)** The lysates from adult mouse testis, germ cells and Sertoli cells were subjected to immunoblot analysis for SENP3 with GAPDH as a loading control. Each bar in the graph was presented as mean ±SD of at least three independent experiments. **(D)** Expression of SENP3 in seminiferous epithelium. Sections of adult mouse testis were prepared for determination of SENP3 localization by immunohistochemistry analysis with anti-SENP3 antibody with rabbit IgG as the negative control. Sertoli cell (black arrow), spermatocytes (red arrow), round spermatids (yellow arrow) and sperm (blue arrow). Scale bar, 25μm. **(E)** Representative images showing the intracellular localization of SENP3 in Sertoli cells for SENP3 (red), α-Tubulin (green) and DAPI (DNA, blue) immunofluorescence staining. Scale bars: 20 μm.

Sertoli cells maintain a mitotically quiescent state *in vivo* after puberty to execute its destined mission to support spermatogenesis [1]. To uncover the potential relevance of SENP3 to Setoli cell function, we confirmed the expression of SENP3 in Sertoli cells. RT-PCR analysis using primers specific to *senp3* reveled *senp3* mRNA expression in adult testis, germ cells as well as Sertoli cells with S16 severs as the loading control (Figure 1A). The specificity of SENP3 antibody was validated by immunoblot assay of Sertoli cells extracts (Figure 1B), and SENP3 protein expression in testis was further confirmed by relative quantitative immunoblot analysis (Figure 1C). Cell purity were determined by semi-quantitative RT-PCR with specific markers such as c-Kit receptor, fibronectin, 3β-HSD and testin, which correspond to germ, peritubular myoid, Leydig and Sertoli cells, respectively (Supplementary Figure 1). Of note, the enrichment of SENP3 in the nucleus of Sertoli cells (Figure 1D, black arrow) and spermatocytes in adult testis was observed by IHC (Figure 1D, red arrow). Moreover, SENP3 predominantly localized in nucleolus at interphase, which is reminiscent of the canonical nucleolus-localized signals of SENP3 reported in mitotic cells (Figure 1E). To deeper explore the expression dynamics of SENP3 in Sertoli cells, its expression at different cell cycle stages was then determined. Once the cells step into M phase, SENP3 signals diffused into cytoplasm and the cytoplasm-diffused signals persisted until early telophase. More significantly, SENP3 re-accumulated into the newly reforming nucleus at late telophase and re-appeared in nucleolus at interphase afterwards (Supplementary Figure 2). Based on its expression dynamics in Sertoli cells, we postulate that SENP3 might potentially play a role in maintaining its function.

### SENP3 knockdown alters the dynamic of SUMO-2/3 conjugates in Sertoli cells

**Figure 2:**
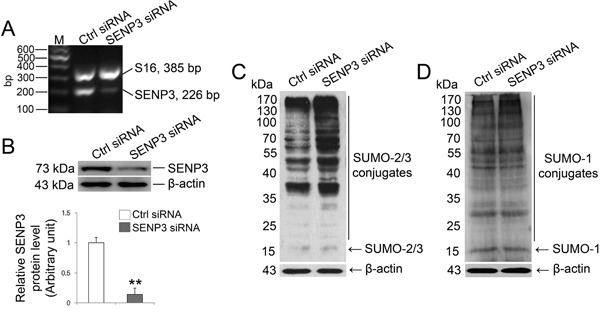
SENP3 depletion intervene SUMO-2/3-ylation profile in Sertoli cells Sertoli cells cultured for 3 d were processed to introduction of SENP3 siRNA or control siRNA at 100 nM, respectively, for 48 h prior to further assays. **(A)** Expression of SENP3 in Sertoli cells determined by RT-PCR analysis using primer specific to *SENP3* with S16 served as the loading control. **(B)** Sertoli cells were lysed for determination of SENP3 expression by immunoblot analysis with anti-SENP3 antibody. Relative band intensity normalized to β-actin was shown in the below panel. SUMO2/3-ylation **(C)** and SUMO1-ylation **(D)** dynamics were detected in Sertoli cells. Each bar in the graph was presented as mean ±SD of at least three independent experiments. *, p< 0.05; **, p< 0.01.

SENP3 has a high propensity to release SUMO-2/3 monomer from its targets [18] which promoted us to determine the effect of SENP3 on SUMOylation profile in Sertoli cells. To this end, we knocked down SENP3 by introducing siRNA against *Senp3* into Sertoli cells, which yielded ~80% of reduction in both mRNA and protein levels (Figure 2A and 2B). The abundance of other SENPs was not altered by SENP3 knockdown, which confirmed the specificity of *Senp3* siRNA (Supplementary Figure 3). Consistent with previous reports, we found that SUMO-2/3 but not SUMO-1 conjugates were significantly augmented after SENP3 knockdown (Figure 2C and 2D). Overexpression of SENP3 decreased the global SUMO-2/3 conjugations (Supplementary Figure 4). Taken together, our results indicate that SENP3 is mainly responsible for de-conjugating SUMO-2/3 from targets while its hydrolase activity to processing of SUMO-2/3 precursor in Sertoli cells could be disregarded to some extents in this specific case.

### SENP3 knockdown disrupts the TJ permeability barrier

**Figure 3:**
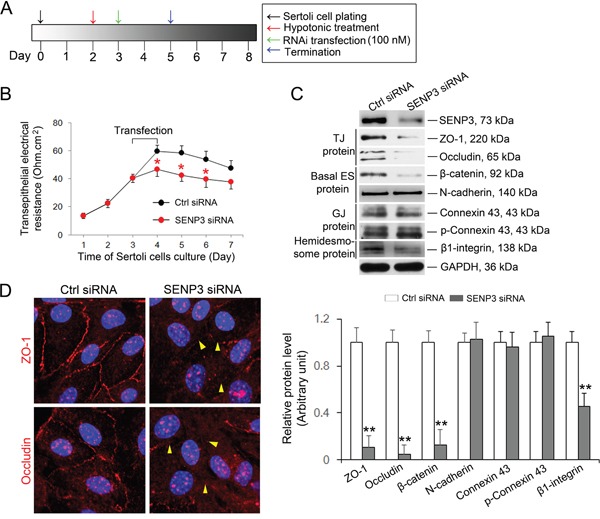
SENP3 depletion compromises Sertoli cell BTB function **(A)** Schematic representation of the regime used *in vitro* assessment of BTB function. **(B)** Graph showing the integrity of TJ permeability barrier. Control and SENP3-depleted Sertoli cells processed as in A were prepared prior to determination of TJ permeability barrier function by trans-epithelial electrical resistance (TER) detection. The TER was detected up to 7 d. Date was presented as mean ± SD of 5 independent replicates. **(C)** Sertoli cells depleted for SENP3 were prepared for immunoblot analysis of those factors required for BTB function, such as TJ proteins (ZO-1 and occludin), basal ES protein (β-catenin and N-cadherin), GJ protein (Connexin 43 and p-Connexin 43), and Hemidesome protein (β1-integrin). The relative abundances normalized to GAPDH was shown in the below panel. Of note, SENP3 depletion (~80%) by introducing 100 nM *senp3* siRNA incites significant decrease in TJ protein as well as in basal ES protein, while the abundances of GJ protein remain unchanged. **(D)** Representative confocal Z-projections showing the distribution of TJ protein at cell-cell interface. Sertoli cells cultured for 2 d were transfected with control or *senp3* siRNA. Two days thereafter, Sertoli cells were fixed and processed for immunofluorescence staining of ZO-1/occludin (red) and DAPI (DNA, blue). Notably, disruption of either ZO-1 or occludin (indicated by yellow triangle) at cell-cell interface was witnessed by SENP3 depletion. Scale bars: 20 μm. *, p< 0.05; **, p< 0.01.

Based on the expression of SENP3 in Sertoli cells, we sought to investigate whether SENP3 has relevance to regulate BTB integrity. Primary Sertoli cells after 2-3 days of culturing are known to form a functional TJ barrier with ultrastructures of TJ, basal ES, GJ and desmosome that mimics the BTB *in vivo* [1]. On day 3, *Senp3* siRNA or control siRNA duplex was transfected into Sertoli cells for 24 h, and then the transfection mixture was removed followed by another 24 h of culturing prior to further analysis according to the regimen (Figure 3A). Knockdown of SENP3 in Sertoli cells was found to induce a disruption of the TJ barrier function evidenced by the precipitous decrease in trans-epithelial electrical resistance (TER) when compared with that in control cells (Figure 3B). Additionally, conspicuous decline in the abundances of TJ proteins (e.g., ZO-1, occludin), basal ES proteins (e.g., β-Catenin) and hemidesmosome protein (e.g., β1-integrin) were induced without affecting the levels of gap junction proteins (e.g., Cx43 and p-Cx43) after depletion ~80% of SENP3 (Figure 3C). Furthermore, the cell interface distribution of TJ proteins ZO-1 and occludin were examined. The ring-shaped bight signals of ZO-1 and occludin were observed at interface in control cells, while the signals of ZO-1 and occludin were withered and mis-located at the cell interface in SENP3-depleted Sertoli cells (Figure 3D, right, yellow arrow), further supporting the loss of occludin and ZO-1 was indeed triggered by SENP3 knockdown. The aforementioned results demonstrated that a regulatory role of SENP3 by affecting distribution of TJ and basal ES, but not GJ proteins at the BTB.

### Knockdown of SENP3 compromises STAT3 transcriptional activity

**Figure 4:**
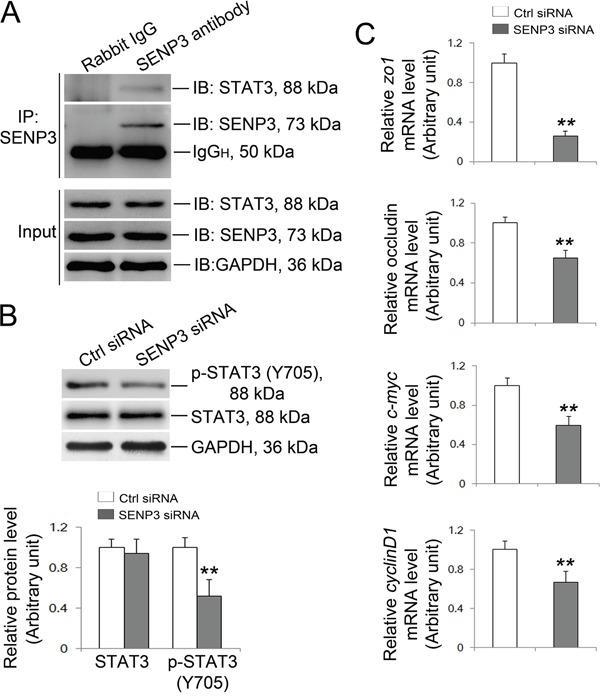
SENP3 silencing wakens the transcriptional activity of STAT3 **(A)** Endogenous SENP3/STAT3 interaction was determined by co-IP in Sertoli cells. **(B)** Immunoblot analysis of p-STAT3 and total STAT3 in control and SENP3-depleted Sertoli cells. Relative bands intensities normalized to GAPDH are as shown in the right panel. **(C)** Graphs showing the relative mRNA level of STAT3 target genes *zo1*, *occludin*, *c-Myc* and *cyclinD1* were determined by qRT-PCR at 48 h post transfection, and normalized to S16 by 2^−ΔΔCT^ method. Each bar in the histogram is a mean ±SD of n=3 experiments. *, p< 0.05; **, p< 0.01.

The transcription factor STAT3 which binds to the putative binding sites in promoter regions of *zo-1* and *occludin* has recently been recognized as target of SUMO-2/3 [22]. More importantly, SENP3 could boost phosphorylation of STAT3 by alleviating SUMO-2/3-ylation of STAT3 and thereby enhances its transcriptional activity [21], arising the possibility that repressed transcriptional activity of STAT3 could be a priori of the observed down-regulation of ZO-1 and occludin in SENP3 depleted Sertoli cells. The Co-IP based on the endogenous setting of Sertoli cells showed that SENP3 indeed interacted with STAT3 (Figure 4A). For this, we then determined the transcriptional activity of STAT3 by examining its phosphorylation at Y705. As anticipated, Y705 phosphorylation of STAT3 was significantly decreased while in the absence of affecting its total level by SENP3 knockdown (Figure 4B). Moreover, the mRNA levels of its known target genes such as *c-myc*, *cyclinD1*, *zo-1* and *occludin* were all significantly repressed by SENP3 knockdown by using qRT-PCR analysis (Figure 4C). Notably, overexpression of SENP3 could promote p-STAT3 level (Supplementary Figure 5A). It could be inferred that the decreased STAT3 transcriptional activity might emerge as the major contributor to the defective TJ permeability caused by SENP3 knockdown in Sertoli cells.

### Loss of SENP3 attenuates F-actin organization

**Figure 5:**
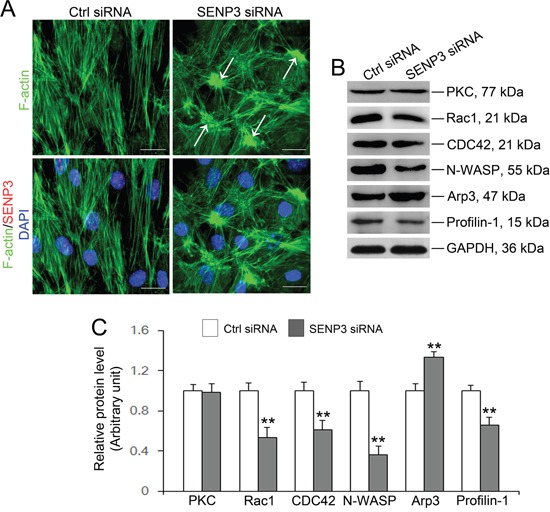
SENP3 knockdown disrupts actin microfilaments reorganization in Sertoli cells by dysfunction of Arp3 and Profilin-1 **(A)** Effects of SENP3 on the actin cytoskeleton in Sertoli cells. Sertoli cells cultured for 2 d were transfected with control or *Senp3* siRNA. Two days thereafter, Sertoli cells were fixed and processed for FITC-phalloidin (F-actin, green) staining or immunofluorescence staining of SENP3 (red). DNA was visualized with DAPI (blue) staining. Significantly, Sertoli cells depleted for SENP3 were prominently featured by disorganized actin microfilaments with dense clusters (annotated by white asterisk). **(B)** Immunoblot analysis of selected regulators of actin dynamics as indicated, and the relative bands intensities normalized to GAPDH were shown in **(C)**. Notably, up-regulation of Arp3 and down-regulation of Rac1, CDC42, N-WASP, and Profilin-1 were observed by SENP3 depletion. Scale bars: 20 μm. Each bar in the histogram is a mean ±SD of n=3 experiments. *, p< 0.05; **, p< 0.01.

Spermatogenesis is largely predefined by the actin-based cytoskeleton architecture, which has been reported to be dynamically regulated by SUMOylation [24, 25, 26, 28]. To pinpoint whether destruction of TJ barrier function in SENP3-depleted Sertoli cells could be ascribe to disrupted F-actin organization, F-actin dynamic was therefore analyzed. Expectedly, SENP3 knockdown was found to have profound effects on F-actin microfilaments. In SENP3-depleted Sertoli cells, F-actin was highly clustered and disorganized, and the microfilaments failed to stretch across the cell but were highly centralized in cell cytosol and become more thick at some parts near the nucleus (white arrow) (Figure 5A). To uncover the underlying mechanism by which SENP3 dictates F-actin dynamics, the function of several actin regulating proteins were then investigated. This plausibly due to robustly expressed Arp3 which initiates branching on preexisting actin filaments to facilitate junction restructuring and cell movement [10], and decreased Profilin-1 (Figure 5B and 5C). Notably, Profilin-1 antagonized Arp2/3 complex to maintain actin homeostasis by balancing Arp2/3 complex and assists in F-actin assembly [29]. Small GTPases of the Rho family are major regulators of actin cytoskeleton as Rac1 and CDC42 are the upstream activators of N-WASP which binds to Arp2/3 to facilitate actin branching [12, 30]. The expressions of Rac1/CDC42/N-WASP components were abruptly decreased by SENP3 knockdown, without affecting the levels of PKC (Figure 5B and 5C). Significantly, the remarkable increase in Rac1, CDC42 and Arp3 abundance was reversibly observed by SENP3 overexpression, while the level of N-WASP was not apparently influenced (Supplementary Figure 5B). Overall, our results indicate that F-actin organization is dictated by Rac1/CDC42-N-WASP-Arp2/3 axis and Profilin-1, the disruption of which may lead to loss of cell polarity by disorganizing F-actin in SENP3-depleted Sertoli cells.

### Knockdown of SENP3 attenuates Elk1 transcriptional activity

**Figure 6:**
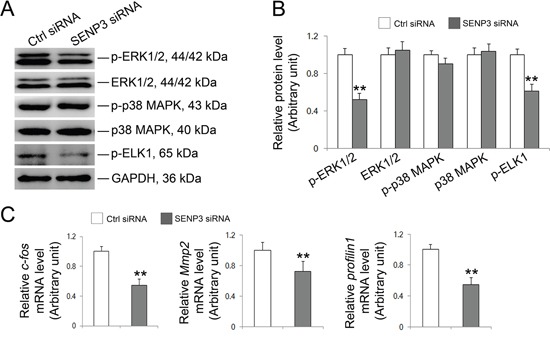
SENP3 involves in MAPK signaling pathway in Sertoli cells Sertoli cells cultured for 3 d were transfected with 100 nM *senp3* siRNA or control siRNA for 48 h followed by further analysis. (**A** and **B**) Immunoblot analysis of the well-known components in MAPK signaling pathways were indicated **(A)**, and their relative abundances normalized to GAPDH were shown in **(B)**. **(C)** Graphs shown were the relative mRNA levels of p-Elk1 target gene *c-fos*, *Mmp2* and *profilin1* by qRT-PCR analysis at 48 h post transfection, and normalized to S16 by 2^−ΔΔCT^ method. Each bar was presented as mean ±SD of n=3 experiments. *, p< 0.05; **, p< 0.01.

The significance of MAPK signaling has been well documented in regulating BTB dynamics and the adhesion of Sertoli cells with germ cells [5, 6, 31]. MAPK signaling recently has been coupled with SUMOylation as SUMOylation could orchestrate the activities of a wide spectrum of transcription factors including Elk1 [32, 33, 34, 35]. De-SUMOylation is a prerequisite for Elk and ERK activation by unmasking the Ser383 phosphorylation site on Elk1 and facilitating the interaction between MEK and ERK, respectively [32, 34]. To decipher whether MAPK pathway was affected in SENP3-depleted Sertoli cells, proteins that related to MAPK signaling were then evaluated. Notably, a precipitous decrease in phospho-ERK1/2 abundance with unaltered phospho-p38 level was incites by SENP3 depletion while the total abundances of ERK and p38 remained unchanged (Figure 6A). Furthermore, the phosphorylation of Elk1, a putative target of p-ERK1/2, was significantly decreased (Figure 6B), implying that the kinase activity of p-ERK1/2 was interfered. Phosphorylation endows Elk1 with the transcriptional activity, arising the possibility that if the disrupted expression of Profilin-1 (Figure 5C) might due to the decreased Elk1 transcriptional activity in SENP3-depleted Sertoli cells. To this end, we determined the abundances of some of Elk1 target genes including Profilin-1. As anticipated, the mRNA level of *Profilin*-1, *c-fos* and *Mmp*2 were significantly declined (Figure 6C), further confirming the deterioration of Profilin-1 was due to the weakened Elk1 transcriptional activity caused by compromised Elk1 phosphorylation. Overall, we contend that the disrupted F-actin architecture could be partially ascribe to the attenuated Elk1 transcriptional activity which down-regulates Profilin-1 in SENP3-depleted Sertoli cells.

## DISCUSSION

Currently, male infertility has been becoming more and more prevalent around the world, which highlights the importance of understanding the molecular mechanisms that govern the permissive microenvironment for spermatogenesis. Our study for the first time deciphers the functional relevance of SENP3 in BTB dynamic regulation by dictating TJ integrity and actin architecture in mouse Sertoli cells which defines SENP3 as a novel determinant in mammalian spermatogenesis program.

Previous studies have shown that SUMO-1 and SUMO-2/3 participate in human spermatogenesis, and the high expression of SUMO-2 is also observed in rat Sertoli cells [17, 36]. SENP3 regulates intracellular processes mainly by removing SUMO-2/3 from specific targets including multiple transcription factors or co-factors such as HIF-1α, p300 and STAT3 [20, 21]. Here, we found that SENP3 localized in Sertoli and spermatocyte cells near the BTB. More importantly, SENP3 knockdown impaired the tight junction permeability accompanied by a decline in trans-epithelial membrane resistance in Sertoli cells as revealed by the vitro Sertoli cell system, which mimics the *in vivo* BTB dynamics, due to the significant reduction in or mis-localization of ZO-1 and occludin (the key constituents of TJ) at the cell-cell interface. The transcriptional activity of STAT3, an important mediator of cytokine signaling for oncogenesis and cancer cell migration, is mainly depends on post-translational modifications [21, 37]. SUMO-2/3 attaches to STAT3 at K451 and negatively regulates its activity by restricting its phosphorylation at Y705. Significantly, its inhibitory phosphorylation at Y705 could be ameliorated by SENP3 which releases STAT3 from the SUMO-2/3 conjugated form [21]. STAT3 binds to the putative binding sites in promoter regions of *zo1* and *occludin* and thereby regulates their expression [22]. Of note, we found that SENP3 interacted with STAT3 under normal condition and enhanced STAT phosphorylation (Figure 4). The phosphorylation, nuclear import and function of STAT3 are regulated by Rac1/CDC42 [38]. We could not exclude the possibility that the striking decrease in Rac1 and CDC42 might also potentiate STAT3 inactivation in SENP3-depleted Sertoli cells. Therefore, we conclude that SENP3 emerges as an important regulator of the TJ permeability function through potentiating the transcriptional activity of STAT3 and thereby governs BTB dynamics. Nevertheless, *in vivo* investigations are still much needed to achieve a better appreciation of SENP3 in spermatogenesis.

In Sertoli cells, the recruitment, localization and endocyticvesicle-mediated trafficking of proteins are executed in a cytoskeleton-dependent way during spermatogenesis [23], indicating that actin microfilaments at the basal ES are crucial regulators of BTB dynamics by maintaining tight junction integrity in Sertoli cells. A wealth of document has shed light on the importance of SUMOylation in dictating actin dynamics by attaching SUMO isoforms to multiple actin regulators including Rac1, Coronin 2A [24, 28, 39]. Rac1 and CDC42 belong to the Rho GTPase subfamily and regulate TJ integrity by interfering with actin organization and cadherin recruitment to adherens junction [23, 40]. Moreover, the function of N-WASP and Arp2/3 are regulated by CDC42, facilitate actin polymerization by generating branched-actin arrays to remodel BTB through endocytic recycling of BTB components [10, 12]. Significantly, we found that F-actin microfilaments were disarranged showing that F-actin unable to stretch across the cell cytosol and become thicker in some areas in SENP3 depleted Sertoli cells. Furthermore, TJ proteins (e.g. ZO-1 and occludin) were no longer properly enriched at the Sertoli cell-cell interface, and these components may be degraded by proteasome through endocytic recycling. Together with the increase in Arp2/3 and the deterioration of Profilin-1, the expression of actin regulatory proteins Apr2/3 and N-WASP as well as their upstream regulators, GTPases Rac1 and CDC42, were precipitously disrupted, which may lead to BTB restructuring in SENP3-depleted Sertoli cells [10]. SUMO E3-ligase PIAS3 interacts with Rac1 and is required for Rac1 activation and optimal cell migration [25]. Therefore, SENP3 might also participate in the SUMOylation dynamics of these GTPases. Overall, our study demonstrates that SENP3 is crucial to cytoskeleton dynamics presumably through its ability to elaborately modulate the expression of GTPase Rac1 and CDC42, and the actin-binding protein Arp2/3.

Another relevant finding seen following SENP3 knockdown is the disruption of MAPK signaling pathway. MAPK signaling is critical to spermatogenesis, which involves in mitotic renewal of spermatogonia, cell division, junction restructuring and cytoskeleton structure [41]. Moreover, cytokines (e.g., TNFα and TGF-β3) and environmental toxicants (e.g., cadmium and BPA) disrupt TJ barrier function also via activating MAPK cascade [5, 31, 42, 43]. In this study, the destruction in ERK1/2 phosphorylation was likewise witnessed in SENP3-depleted Sertoli cells. Previous studies have demonstrated that the activities of multiple transcription factors are dictated by SUMO pathway, and the SUMO pathway has also been linked to MAPK signaling [32]. MEK SUMOylation blocks ERK activation by disrupting the specific docking interaction between MEK and ERK, and Ras could efficiently activates the ERK pathway by inhibiting MEK SUMOylation [44]. Thus, the hyperSUMOylation due to imbalanced SUMOylation dynamic caused by SENP3 depletion may explain the dampened ERK activity in Sertoli cells. Another important finding is the transcriptional activity of p-Elk1 was repressed. The Elk1 target gene such as *PTGS2*, *CTGF*, *HIF1A*, *PFN1*, *IQGAP*-1, and *LIMA*-1 are closely related with actin dynamic and cell migration [45]. Among them, mRNA and protein level of Profilin-1, which serves as a gatekeeper for actin assembly by balancing Arp2/3 complex [29], were declined in SENP3-depleted Sertoli cells, which might obstruct actin polymerization. SUMOylation represses the activities of Elk-1 and the co-activator of Sp1 and HIF-1α, p300, and these inhibitory effects could be reversed by the deSUMOylation activity of SENP3 [20, 33]. The status of p300 as the co-activator of ELK1 may also effect its transcriptional activation [46], raising the possibility that SUMOylation might also regulate ERK activity through the p300 branching pathway. Despite the globe increase in SUMO-2/3-ylation could explain how ELK1 activity is repressed in SENP3-depleted Sertoli cells to some extents, further studies are still need to decipher the detailed basic mechanisms by which SENP3 dictates Elk1activity.

**Figure 7:**
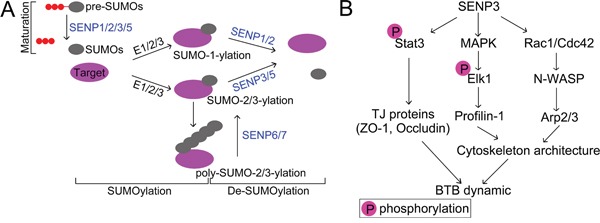
SENP3 involves in MAPK signaling pathway in Sertoli cells **(A)** Schematic diagram of SUMO pathway. Post-translational modification of proteins with small ubiquitin-like modifier (SUMO) is a highly dynamic process. SUMO precursors are processed by the corresponding Sentrin/SUMO specific proteases which possess endopeptidase activity (SENP1/2/3/5) to become mature. Conjugation of SUMO to its substrate is programmed by a series of enzymatic cascades catalyzed by E1 activating enzymes (a heterodimeric, Aosl/uba2), E2 conjugating enzyme Ubc9, and E3 ligating enzymes. SUMO2/3 contains the SUMO consensus modification motif (ψKxE/D) (ψ is a hydrophobic amino acid and x is any amino acid) which allows the formation of poly-SUMO chains [50]. Removal of SUMO from substrate is likewise executed by SUMO specific proteases which possess isopeptidase activity with SENP1/2 has the propensity of releasing SUMO1 rather than SUMO2/3, and SENP3/5 preferentially deconjugates SUMO2/3. While the poly-SUMO chains are specifically edited SENP-6/7 [51]. **(B)** Schematic representation of the relevance of SENP3 in regulating BTB dynamic. SENP3 modulates the transcriptional activity of STAT3 which dictates the expression of those genes involved in tight junction, such as ZO-1 and Occludin. Furthermore, SENP3 also orchestrate cytoskeleton architecture through interfering with Rac1/Cdc42-N-WASP-Arp2/3 pathway, and MAPK-Elk1 signaling, which licenses the expression of Profilin-1. Depletion of SENP3 therefore incites the dysfunction of those regulators required for tight junction integrity and cytoskeleton architecture, respectively, in Sertoli cells, and thereby undermines the microenvironment for proper spermatogenesis maintained by BTB dynamic.

In conclusion, our study dispels the myth of SENP3 in Sertoli cells biology by determining its functional relevance to tight junction permeability and F-actin organization, which further buttresses the pivotal role of SUMOylation in maintaining a proper microenvironment for spermatogenesis (Figure 7). These findings will broaden our knowledge of the molecular machinery regulating BTB dynamics in mammals but also might provide important implications for the development of clinic therapeutic for male infertility.

## MATERIALS AND METHODS

### Animals and ethnics statement

The present study was approved by the Ethical Committee of Hubei Research Center of Experimental Animals (Approval ID: SCXK (Hubei) 20080005). Wild-type Kunming (KM) mice were obtained from the local Central Animal Laboratory and housed in the experimental animal center of Huazhong Agricultural University under a 12 h light/12 h dark regimen at a temperature of 22°C with water and food ad libitum. All experimental procedures were performed in line with the guidelines of the Committee of Animal Research Institute, Huazhong Agricultural University, China.

### Germ cells and primary Sertoli cell isolation

Germ cell were isolated from adult mice testes, cultured in F12/DMEM supplemented with sodium lactate and sodium pyruvate, and prepared for protein extraction within 12 hours with a cell viability > 95% [47]. Primary Sertoli cells were isolated from 3 weeks old male mice testes and cultured in serum-free F12/DMEM supplemented with EGF, bovine insulin, human transferrin and bacitracin at 35°C with a humidified atmosphere of 5% CO_2_ in air as described [48]. Freshly isolated Sertoli cells were seeded on Matrigel-coated (Corning): (i) 6- or 12-well dishes at 0.5×10^6^ cells per cm^2^ were subsequently used for lysate preparation, (ii) coated coverslips at 0.04-0.08×10^6^ cells per cm^2^ were processed to immunofluorescent analysis. Cells were cultured for 36 h prior to hypotonic treatment using 20 mM Tris, pH 7.4, at 22°C for 2.5 minutes to lyse residual germ cells. Cell purity was assessed by semi-quantitative RT-PCR against specific markers such as c-Kit receptor, fibronectin, 3β-HSD and testin, corresponding to germ, peritubular myoid, Leydig, and Sertoli cell, respectively [49].

### SENP3 knockdown in Sertoli cells

Primary Sertoli cell obtained from testes of 3 weeks old mice testes were cultured for 3 days, and then subjected to transfection with 100 nM non-targeting or *Senp3*-specific siRNA duplexes for 24 hours by RNAi MAX Transfection Reagent according to the manual (Thermo Scientific, Waltham, MA). After transfection, the reaction mixture was removed and replaced with fresh F12/DMEM, and cells were cultured for another 24 h before termination for immunofluorescent analysis (IF) or for RNA extraction and lysate preparation. The *Senp3*-specific siRNA (sc-45718; Santa Cruz, CA) and non-targeting siRNA (sc-37007; Santa Cruz, CA) control duplexes were purchased from Santa Cruz and the sequences are shown in Supplementary Table 1.

### Functional assessment of the Sertoli cell TJ permeability barrier *in vitro* by trans-epithelial electrical resistance (TER) measurement

Primary Sertoli cell obtained from testes of 3 weeks old mice were plated on Millicell bicameral units (diameter, 12 mm; pore size, 0.45 μm, effective surface area, ~ 0.6 cm^2^; Millipore) at 1.2×10^6^ cells per cm^2^ placed in 24-well dishes containing 0.5 ml F12/DMEM to assess the Sertoli cell TJ permeability barrier function by quantifying trans-epithelial electrical resistance (TER) across the cell epithelium. On day 3, Sertoli cell were introduced with 150 nM *Senp3*-specific and non-targeting control duplexes with RNAi MAX Transfection Reagent. The TER was assessed every 12 h until day 7 and each date was presented as mean ± SD of n = 5 replicates as described. A total of four direction positions should be recorded for each bicameral culture unit. The true TER value was calculated as TER _sample_ (Ωcm^2^) = (Resistance _sample_-Resistance _blank_) (Ω) × Effective surface area (cm^2^) described by Mruk and Cheng [48].

### Immunohistochemistry (IHC) and immunofluorescent (IF) analysis

Immunohistochemistry was performed as previously described [9]. Briefly, testes was fixed with 4% paraformaldehyde in PBS for 24 h at 4°C, embedded in paraffin, sectioned to 5 μm and mounted on glass slides. Sections were then deparaffinized and rehydrated, and hated in 10 mM sodium citrate buffer for antigen retrieval followed by elimination of endogenous peroxidase activity in 3% H_2_O_2_ for 30 min. Afterwards, slides were blocked with 10% normal goat serum for 1 h and incubated with respective primary and secondary antibody. Finally, the protein residues were visualized with 3, 3-diaminobenzidine according to the manual, and nucleus was counterstained with hematoxylin. For immunofluorescent or double- immunofluorescent analysis, Sertoli cells cultured at 0.04 × 10^6^ cells per cm^2^ on coverslips were fixed with 4% paraformaldehyde in PBS for 10 min and then permeabilized using 0.1% Triton X-100 for 4 min. Cells were then blocked with 5% BSA for 30 min, followed by an overnight incubation of primary antibodies. After washing in PBS/0.1% Tween/0.01% Triton X-100, cells were processed to incubation with corresponding secondary antibodies at 4°C for 16 h and 37°C for 2 h, respectively. For F-actin staining, Sertoli cells were incubated with FITC-conjugated phalloidin (Sigma, MO). DNA was visualized with DAPI (10 μg/ml) for 15 min at room temperature and then coverslips were mounted to slides with DABCO followed by examination with confocal laser scanning microscope (ZEISS LSM 510 META, Carl Zeiss Imaging, Germany) equipped with a Plan-Apochromat 63 × /1.4 oil DIC objective. Confocal images were processed using Zeiss LSM Image Browser software and Adobe Photoshop (Adobe Systems Inc., San Jose, CA). For the negative control, non-immunized rabbit or goat IgG were used to replace the primary antibodies. Antibodies and dilutions were listed in Supplementary Table 2.

### Immunoblot and real-time qPCR analysis

Sertoli cell extracts were prepared by lysing cells in extraction buffer (50 mM Tris, 150 mM NaCl, 1% Triton X-100, 1% sodium deoxycholate, 0.1% SDS, pH 7.4) supplemented with protease and phosphatase inhibitor mixtures and PMSF (Sigma, MO), sonicating, and centrifuging at 14,000 × g for 15 min to obtain the clear supernatant. Lysates were stored at -80°C until used. For immunoblot analysis, lysates (30 μg of protein) were separated by SDS/PAGE gel, transferred to PVDF membrane (Immobilon-P; Millipore), and blocked in TBS/0.1% Tween-20/5% BSA (TBS, 25 mM Tris, 150 mM NaCl, pH 7.4) for 1 h, followed by primary antibodies incubation for overnight at 4°C. After washing in TBS/0.1% Tween-20, membranes were subjected to incubation with corresponding HRP-conjugated secondary antibodies for 1 h at room temperature. The immunoblot bands were visualized with ECL kit and read using chemiluminescence system (Thermo Scientific, Waltham, MA). For quantitative analysis, the immunoblot protein bands were assayed by the ChemiDoc XRS Imaging System (Bio-Rad). Beta-actin or GAPDH was served as the loading control and the relative signal intensity was assessed by ImageJ software (NIH, USA). For negative control, the non-immunized rabbit or mouse IgG were used to replace the primary antibodies. Antibodies and dilutions were listed in Supplementary Table 2.

RNA from cells and tissues was performed using TransZol reagent (TRANSGEN, BeiJing) according to the manufacturer's instructions. For reverse transcription using RevertAid First Strand cDNA Kit (Thermo Scientific, Waltham, MA) to obtain cDNAs and amplification by PCR using specific primer pairs. The authenticity of PCR products was verified by direct DNA sequencing analysis at TSINGKE (TSINGKE Biological Technology, BeiJing). Fluorescence real-time qPCR was performed with QuantiFast SYBR Green PCR Master Mixes (QIAGEN Hilden, Düsseldorf) using the CFX96 Touch^TM^ Deep Well Real-Time PCR Detection System (Bio-Red Hercules, CA) with specific primer pairs (Supplementary Table 3). Primer sequence for *senp3* expression vector is decribed in Supplementary data, Supplementary Table 4.

### Co-immunoprecipitation assay

Lysates from Sertoli cells were harvested with lysis buffer (10 mM Tris, 150 mM NaCl, 1% NP-40, 10% glycerol, pH 7.4) supplemented with protease and phosphatase inhibitor mixtures and PMSF (Sigma, MO). Cell lysates were centrifuged at 14000× g for 15 min at 4°C, followed by immunoprecipitation with anti-SENP3 antibody and rabbit IgG which served as the negative control, respectively, at 4°C for overnight. The lysates were then processed to incubation with protein A/G-Sepharose beads (Beyotime, Shanghai) for 2 hours, and the beads were thereafter washed four times with cold lysis buffer. Proteins were released from the beads by heating at 100°C for 5 min in 2× Laemmli sample buffer. Immunoblot was performed as described above.

### Statistical analysis

Data from at least 3 replicates was presented as mean ± SEM and analyzed by paired-samples t-test using SPSS software (SPSS Inc, Chicago, IL) with p< 0.05 was considered to be statistically significant. Different superscripts indicate the statistical difference.

## SUPPLEMENTARY MATERIALS FIGURES AND TABLES



## Abbreviations

SENP3: sentrin/SUMO-specific proteases 3
BTB: blood-testis barrier
TJ: tight junction
basal ES: basal ectoplasmic specializations
TER: trans-epithelial electrical resistance
