# Supplementary material for: SENP3 grants tight junction integrity and cytoskeleton architecture in mouse Sertoli cells
Source: Oncotarget. 2017 Apr 7;8(35):58430–42. doi: 10.18632/oncotarget.16915 (PMC5601664; doi:10.18632/oncotarget.16915)
Supplement: Supplementary file 1 [file oncotarget-08-58430-s001.pdf]

SENP3 grants tight junction integrity and cytoskeleton architecture in mouse Sertoli cells

SUPPLEMENTARY DATA

Overexpression of SENP3 in Sertoli cell cultures

The full-length coding sequence of WT mouse SENP3 (NM__001163571.1), including start and stop codons, was amplified from mouse Sertoli cell cDNA by PCR using primer listed in Supplementary Table 4, and it was cloned into the Pst I and Xba I sites in the pCMV-N-Flag Vector (Beyotime, Shanghai). The full-length cDNA clone of 1967 bp was confirmed by direct DNA sequencing analysis at TSINGKE (TSINGKE Biological Technology, BeiJing). Plasmid DNA was purified using the Mag-Bind® Ultra-Pure Plasmid DNA Kit (Omega Bio-tek, Norcross, GA). Thereafter, Sertoli cell were transfected with plasmid DNA for 48 h using Lipofectamine® LTX with Plus™ Reagent (Thermo Scientific, Waltham, MA) according to the manufacturer's instructions. Cells were harvested 3 d after transfection for the preparation of lysates.

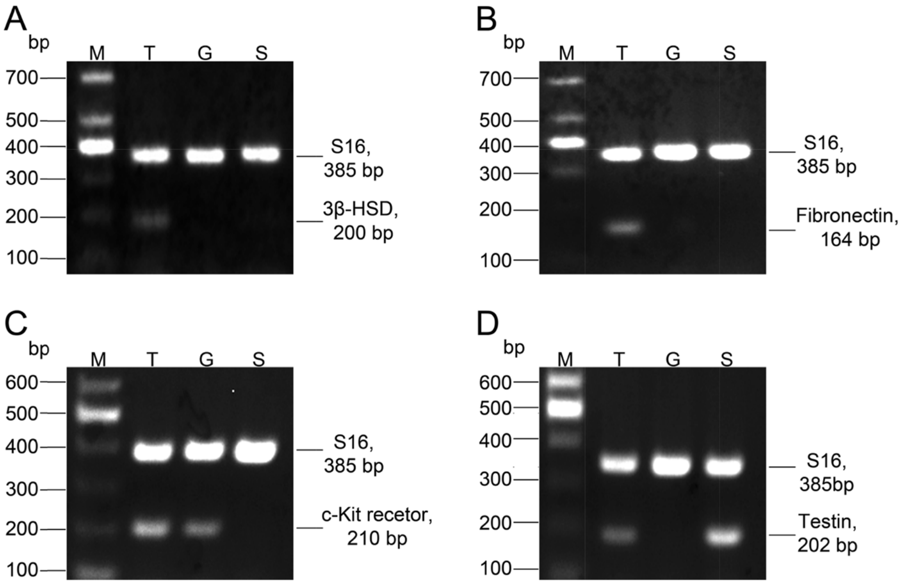

Supplementary Figure 1: Sertoli and germ cell purity were monitored by semi-quantitative RT-PCR in isolated testes (T), germ cells (G) and Sertoli cells (S) using specific primer, and S16 served as a loading PCR control. M, DNA marker in base pair (bp). **(A)** 3β-hydroxysteroid dehydrogenase (3β-HSD) for Leydig cells. **(B)** Fibronectin for peritubular myoid cells. **(C)** c-Kit receptor for germ cells. **(D)** Testin for Sertoli cells. The results demonstrated that the Sertoli and germ cells had negligible other cell types.

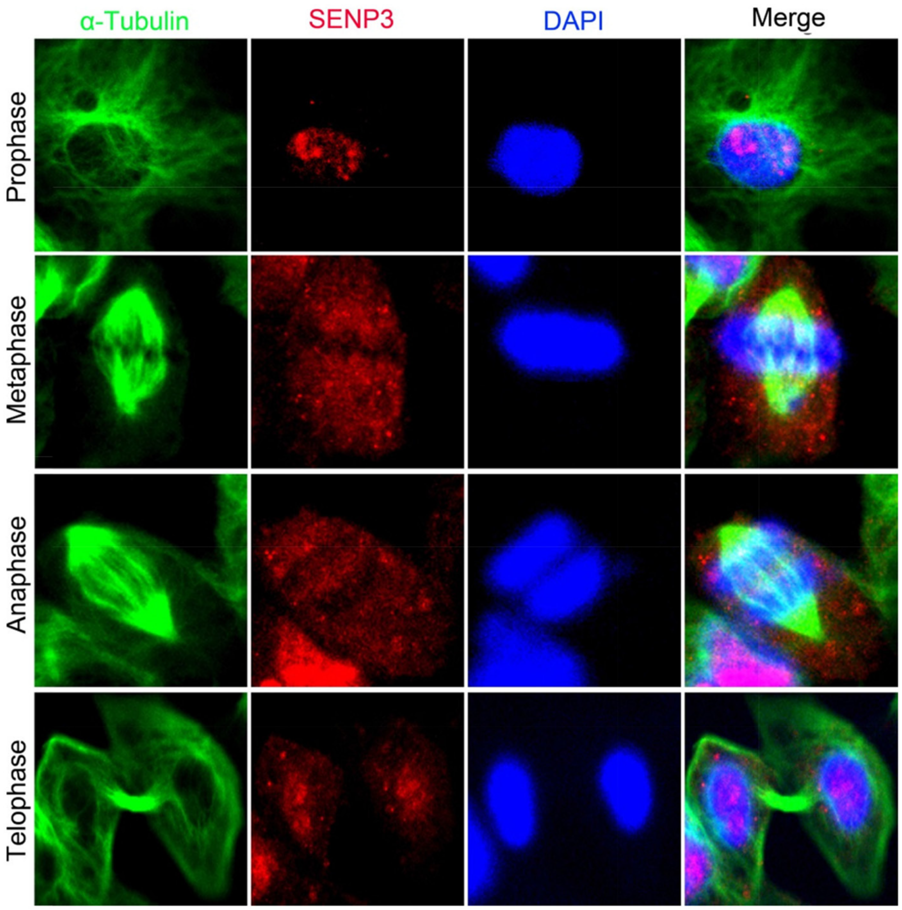

Supplementary Figure 2: SENP3 dynamics in Sertoli cells during different cell cycle stages. Cellular localization of SENP3 was detected by immunofluorescent analysis. Sertoli cells were processed for immunofluorescence staining of anti-FITC-α-Tubulin (green), anti-SENP3 (red) and DAPI (blue).

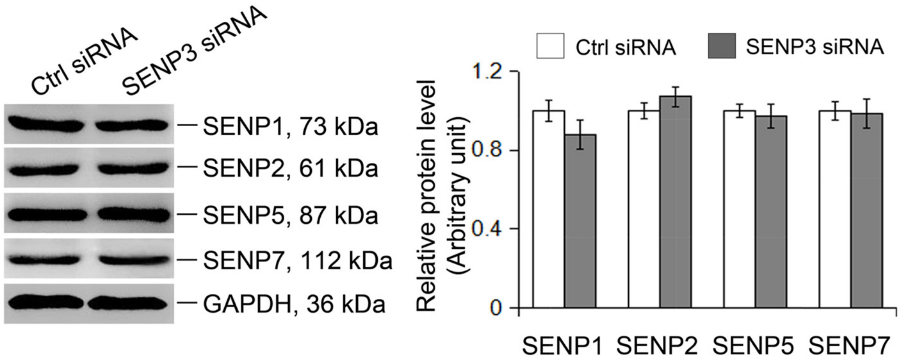

Supplementary Figure 3: Specificity of SENP3 siRNA revealed by immunoblot. Immunoblot showing the steady level of SENP1, SENP2, SENP5 and SENP7 proteins in lysates of Sertoli cells lysed 48 h after SENP3-specific siRNA duplexes and non-targeting control siRNA duplexes transfection; GAPDH served as a protein loading control. Each bar in the histogram is the mean ±SD of n=3 experiments. *, $p < 0.05$; **, $p < 0.01$.

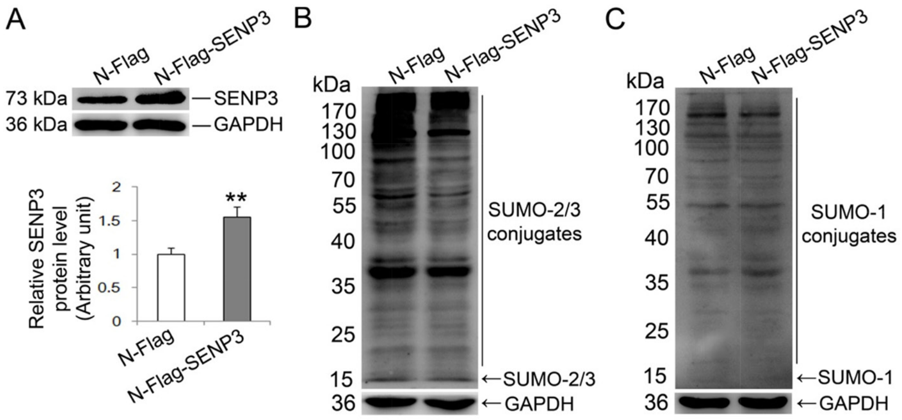

Supplementary Figure 4: Overexpression SENP3 intervene SUMO-2/3-ylation profile in Sertoli cells. Sertoli cells cultured for 3 d were transfected with N-Flag plasmids or N-Flag-SENP3 plasmids for 48 h. **(A)** The efficiency of overexpression of SENP3 was determined by Immunoblot. Relative band intensity normalized to GAPDH was shown in the below panel. SUMO2/3-ylation **(B)** and SUMO1-ylation **(C)** dynamics were detected in Sertoli cells. Each bar in the graph was presented as mean ±SD of at least three independent experiments. *, $p < 0.05$; **, $p < 0.01$.

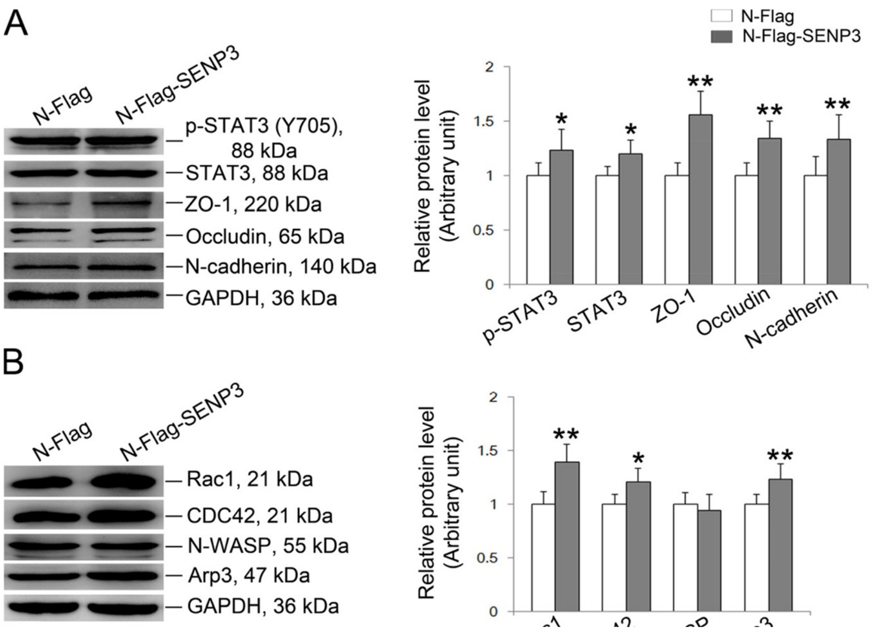

Supplementary Figure 5: Overexpression SENP3 enhances STAT3 phosphorylation and actin regulator proteins. Sertoli cells cultured for 3 d were transfected with N-Flag plasmids or N-Flag-SENP3 plasmids for 48 h. **(A)** Immunoblot analysis p-STAT3, TJ proteins (ZO-1 and Occludin) and basal ES protein (N-cadherin). The relative abundances normalized to GAPDH was shown in the below panel. **(B)** Immunoblot analysis of selected regulators of actin dynamics as indicated, and the relative abundances normalized to GAPDH was shown in the below panel. Each bar in the graph was presented as mean ±SD of at least three independent experiments. *, $p < 0.05$; **, $p < 0.01$.

Supplementary Table 1: The sequences of Senp3-specific siRNA (sc-45718; Santa Cruz, CA)

	Sense/antisense (5'-3')
sc-45718A:889-	GCAUACUCAUCAGUAAUGUTT<br>ACAUUACUGAUGAGUAUGCTT
sc-45718B:1162-	GUCUGGUACUACAGCUGAUTT<br>AUCAGCUGUAGUACCAGACTT
sc-45718C:1388-	CCGUACCAAGGGUUAUGAUTT<br>AUCAUAACCCUUGGUACGGTT

Supplementary Table 2: The information of antibodies used in IF and WB analyses

Product	Catalog	Application and dilution	
		WB	IF
SENP3 antibody	5591, CST	1:1000<br>1:100 (IP)	1:200
SENP3 antibody	GTX117938, GTX		1:500 (IHC)
FITC-α-Tubulin antibody	F2168, Sigma		1:100
SUMO-2/3 antibody	ab109005, Abcam	1:1000	
SUMO-1 antibody	4930, CST	1:1000	
ZO-1 antibody	61-7300, Invitrogen	1:100	1:50
Occludin antibody	71-1500, Invitrogen	1:100	1:50
β-Catenin antibody	8480, CST	1:1000	
N-cadherin antibody	Ab76011, Abcam	1:1000	
Connexin 43 antibody	3512, CST	1:1000	
Phospho-Connexin 43 antibody	3511, CST	1:1000	
Integrin β1 antibody	sc-374429, Santa Cruz	1:500	
PKCαβ antibody	ab179522, Abcam	1:1000	
Phalloidin-FITC	P5282, Sigma		1:100
Rac1/2/3 antibody	2465, CST	1:1000	
CDC42 antibody	ab187643, Abcam	1:1000	
N-WASP antibody	ab126626, Abcam	1:1000	
Arp3 antibody	ab181164, Abcam	1:2000	
Profilin-1 antibody	sc-137235, Santa Cruz	1:500	
44/42 MAPK antibody antibody	4695, CST	1:1000	
Phospho-44/42 MAPK antibody	4370, CST	1:1000	
P38 MAPK antibody	8690, CST	1:1000	
Phospho-P38 MAPK antibody	4511, CST	1:1000	
Phospho-Elk1 antibody	AF3212	1:1000	
Phospho-STAT3 antibody	ab76315, Abcam	1:1000	
STAT3 antibody	ab68153, Abcam	1:1000	
SENP1 antibody	AF0275, Affinity	1:500	
SENP2 antibody	DF8373, Affinity	1:500	
SENP5 antibody	AF0276, Affinity	1:500	
SENP7 antibody	AF0278, Affinity	1:500	
β-Actin antibody	BF0198, Affinity	1:2000	
HRP-GADPH antibody	AB2000, Abways	1:5000	
HRP-goat anti-rabbit antibody	sc2004, Santa Cruz	1:3000	
HRP-goat anti-mouse antibody	sc-2005, Santa Cruz	1:3000	
Cy3-goat anti-rabbit antibody	111-166-045, Jackson		1:100

Supplementary Table 3: The information of primers used for PCR

Gene	Sense/antisense (5'-3')	Extend size (bp)	Reference
Senp3	CAGTCCCTGAAAAGGTGCATTTC<br>GGGCAGCGGCGATTTAGAG	226	
c-fos	GGAGGACCTTACCTGTTCG<br>TTCCAATAATGAACCCAACA	272	
Mmp-2	TGTCCCGAGACCGCTATGT<br>GGCTGCCACGAGGAATAGG	213	
profilin-1	CTGAGGTTGGTGTCCTGGTAG<br>CGTCTTGGCAGTCATGGTGA	191	
c-myc	CTGTATGTGGAGCGGTTTCT<br>GTCGTTGAGCGGGTAGGGA'	208	
cyclin D1	GTGAGGAGCAGAAGTGCGAAGA<br>TGTTCACCAGAAGCAGTTCCATT	237	
occludin	TTCAAACCCAATCATTATGC<br>GCCACTATAAGGGTAGTTTAGG	276	
ZO1	CAAAAAGTGAACCACGAGATG<br>TCAACCGCATTTGGCGTTAC	127	
GAPDH	AGGGCATCTTGGGCTACAC<br>GGTCCAGGGTTTCTTACTCC	210	
S-16	TCCGCTGCAGTCCGTTCAAGTCTT<br>GCCAAACTTCTTGGTTTCGCAGCG	385	[49]

Supplementary Table 4: Primer used for the construction of SENP3 expression vector (NM_001163571.1)

	Primer sequence (5'-3')
Sense	AAAACTGCAGTAGACGCGCTGAGGGTACTG
Antisense	GCTCTAGAGCCACAAGGCAACATTCTC

Sequences marked by underlining represent the restriction enzyme Pst I and Xba I recognition sites added for cloning into pCMV-N-Flag vector.
